# The Novel-B-Cell-Related Gene Signature Predicts the Prognosis and Immune Status of Patients with Esophageal Carcinoma

**DOI:** 10.1007/s12029-024-01083-x

**Published:** 2024-07-04

**Authors:** Xinhong Li, Tongyu Sun, Hongyan Li, Juan Liu, Na Huang, Surong Liu

**Affiliations:** 1 Department of Oncohematology, Norinco General Hospital, Xi’an, Shaanxi, 710061 China; 2 Hepatobiliary and Vascular Surgery, Norinco General Hospital, Xi’an, Shaanxi, 710061 China; 3 Department of Radiology, Norinco General Hospital, Xi’an, Shaanxi, 710061 China; 4 National & Local Joint Engineering Research Center of Biodiagnosis and Biotherapy, the Second Affiliated Hospital of Xi’an Jiaotong University, Xi’an, Shaanxi, 710061 China

**Keywords:** Single-cell RNA sequencing, B-cell marker genes, Immunotherapy, Esophageal carcinoma, Prognostic signature

## Abstract

**Background:**

The current understanding of the prognostic significance of B cells and their role in the tumor microenvironment (TME) in esophageal carcinoma (ESCA) is limited.

**Methods:**

We conducted a screening for B-cell-related genes through the analysis of single-cell transcriptome data. Subsequently, we developed a B-cell-related gene signature (BRGrisk) using LASSO regression analysis. Patients from The Cancer Genome Atlas cohort were divided into a training cohort and a test cohort. Patients were categorized into high- and low-risk groups based on their median BRGrisk scores. The overall survival was assessed using the Kaplan-Meier method, and a nomogram based on BRGrisk was constructed. Immune infiltration profiles between the risk groups were also compared.

**Results:**

The BRGrisk prognostic model indicated significantly worse outcomes for patients with high BRGrisk scores (*p* < 0.001). The BRGrisk-based nomogram exhibited good prognostic performance. Analysis of immune infiltration revealed that patients in the high-BRGrisk group had notably higher levels of immune cell infiltration and were more likely to be in an immunoresponsive state. Enrichment analysis showed a strong correlation between the prognostic gene signature and cancer-related pathways. IC50 results indicated that patients in the low-BRGrisk group were more responsive to common drugs compared to those in the high-BRGrisk group.

**Conclusions:**

This study presents a novel BRGrisk that can be used to stratify the prognosis of ESCA patients and may offer guidance for personalized treatment strategies aimed at improving prognosis.

**Supplementary Information:**

The online version contains supplementary material available at 10.1007/s12029-024-01083-x.

## Introduction

Esophageal carcinoma (ESCA), a highly prevalent gastrointestinal tumor worldwide, is frequently associated with delayed diagnosis and unfavorable outcomes [1, 2]. While surgical intervention remains the primary curative approach for early-stage ESCA, its effectiveness remains limited for patients with advanced disease [3]. Despite advancements in early detection and combination therapies, the 5-year survival rate for ESCA patients remains disappointingly low, hovering around 20% [4, 5]. While certain combination treatment strategies have shown promise in extending survival for individuals with advanced stages [6, 7], their clinical responses continue to be a subject of debate. Accurate prognosis prediction holds paramount significance for effective treatment planning. Hence, there is an urgent need to identify novel biomarkers that can provide insights into the prognosis of ESCA.

The role of the tumor microenvironment (TME) is highly significant in the progression and infiltration of tumors [8]. The TME holds crucial implications for immunotherapy, thus impacting patient survival [9]. The importance of single-cell RNA sequencing (scRNA-seq) in advancing targeted therapy and immunotherapy has gained widespread recognition [10]. Recent advancements in scRNA-seq have revealed distinct subpopulations of immune cells within the TME, presenting a novel approach for characterizing functional biomarkers [11]. Accumulating evidence emphasizes the existence of tumor heterogeneity defined by unique immunosubtypes, with T cells, B cells, natural killer (NK) cells, and infiltrating myeloid cells constituting the predominant components of the tumor immune microenvironment [12–14]. In recent years, the analysis of single-cell transcriptomes has introduced a novel avenue for exploring intra-tumor heterogeneity and predicting interactions within the microenvironment [15, 16]. However, investigations into the immune microenvironment of ESCA have predominantly focused on T cell functionality. In comparison, B cells, the second most prevalent immune cell type, have received relatively less attention in previous research [17]. Therefore, it is essential to gain a comprehensive understanding of the roles played by B cells within the ESCA microenvironment to comprehend the intricate interactions among distinct immunosubtypes, potentially leading to the discovery of innovative therapeutic strategies.

Numerous investigations have aimed to uncover novel cancer biomarkers by integrating scRNA-seq and bulk RNA-seq data [18–20]. In our study, we conducted an integrated analysis of both scRNA-seq and bulk RNA-seq data from ESCA, with the goal of identifying B-cell marker genes and constructing a prognostic signature within the training cohort. To further assess the predictive capacity of this signature, we applied it to the test cohort and the integrated TCGA dataset. Additionally, we scrutinized disparities in the tumor immune microenvironment (TIME) and drug sensitivity across the two risk groups. We hold the belief that our findings have the potential to yield valuable prognostic biomarkers and therapeutic targets for ESCA.

## Materials and Methods

### Data Collection

We retrieved transcript per million (TPM) values for bulk RNA-seq gene expression data and clinical details for ESCA from The Cancer Genome Atlas (TCGA) database (https://portal.gdc.cancer.gov/). Our analysis focused solely on primary solid tumor patients. Additionally, single-cell RNA-seq data (GSE160269) were sourced from the GEO databases (https://www.ncbi.nlm.nih.gov/geo/).

### Identifying B-Cell-Related Genes in ESCA

The Tumor Immune Single Cell Hub 2 (TISCH2) served as a valuable source of single-cell RNA-seq (scRNA-seq) data originating from both human and mouse tumors. This resource facilitated a comprehensive analysis of gene expression within the TME [21]. Our initial step involved extracting B-cell-related genes (BRGs) in GSE160269 analyzed by TISCH2 using specific criteria (|log2FC| > 1 and adjusted *p*-value < 0.05) [22]. We downloaded the expression matrix, UMAP coordinates, sample ID, and cell annotation information from the GSE160269 dataset from TISCH2. Then, we used the “hdf5r” package, “Seurat” package, and “ggplot2” package for data reading and visualization. Subsequently, we conducted an intersection of the genes found in the scRNA-seq GEO dataset and the TCGA dataset. Ultimately, a total of 274 BRGs were identified and compiled from these analyses. We conducted differential analysis of bulk RNA-seq data between cancer and normal samples in the TCGA-ESCA cohort using the “limma” package with |log2FC| > 1 and adjusted *p*-value < 0.05 [23].

### Establishment of a B-Cell-Associated Gene Signature

For the construction and validation of the B-cell-associated gene signature, we randomly partitioned the TCGA-ESCA dataset into training and testing datasets in a 7:3 ratio. To identify genes linked with overall survival (OS) in ESCA patients, we performed a sequential application of univariate Cox regression followed by the least absolute shrinkage and selection operator (LASSO) regression. This methodology enabled us to isolate ten significant B-cell-related genes. Utilizing their expression levels and corresponding regression coefficients, we calculated the risk score as follows: BRGrisk = ∑ regression coefficient (gene i) × gene expression value (gene i). The patient population was then categorized into high-risk and low-risk subsets based on their median risk score values. We subsequently employed Kaplan-Meier (K-M) survival analysis to compare overall survival between these subgroups. We also validated the prognostic gene signature in the entire TCGA-ESCA samples.

### Nomogram and Calibration Assessment

Within the TCGA training dataset, we performed a time-dependent receiver operating characteristic (ROC) curve analysis to assess the prognostic capability of the risk score over various time intervals. Additionally, the nomogram was developed using multivariate Cox regression analysis, integrating both clinical information and the risk score (implemented using the R package “regplot”).

### Functional Enrichment Analysis

We conducted functional enrichment analysis using the GSEA v4.3.2 tool sourced from the MSigDB database (https://www.gsea-msigdb.org/gsea/msigdb) [24]. This analysis aimed to identify significantly associated HALLMARK pathways between the high-risk and low-risk subgroups. The criteria for pathway selection included nominal *p*-value < 0.05.

### Analysis of Tumor Microenvironment and Immune Infiltration Levels

We employed the “estimate” package to assess immune scores, stroma scores, and estimate scores. For the quantification of immune cells and immune function, a single-sample gene set enrichment analysis (ssGSEA) was conducted using the R package “GSVA” [25].

### Drug Sensitivity Analysis

The initial data regarding chemotherapy response were sourced from Genomics of Drug Sensitivity in Cancer (GDSC2) (https://www.cancerrxgene.org/). Curated data were obtained from https://osf.io/temyk. To predict the variance in chemotherapy response between the high-risk and low-risk subgroups, we employed the R package “pRRophetic” [26].

### Statistical Analysis

All statistical analyses were conducted using R software version 4.3.0. A *p*-value < 0.05 was considered statistically significant unless indicated otherwise. Survival analysis was executed utilizing the R packages “survival” and “survminer.” The Wilcoxon test was employed for comparing two groups, while the Kruskal-Wallis test was used for comparing more than two groups.

## Results

### Identification of B-Cell Marker Gene Expression Profiles

**Fig. 1:**
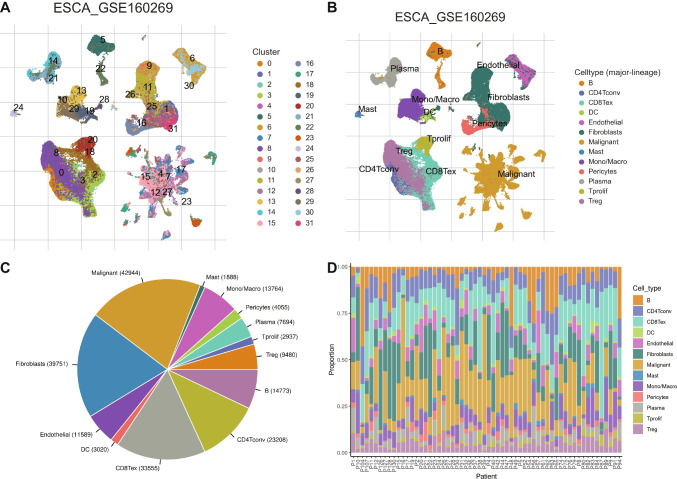
Identification of B cells through scRNA-seq analysis.** A** Thirty-two clusters were discerned through the application of the UMAP algorithm. **B** Thirteen distinct cell types were characterized based on established cell marker genes. **C** The pie chart provides a visual representation of the relative proportions of each defined cell type. **D** The bar plot illustrates the relative distribution of each defined cell type across individual samples

The scRNA-seq data utilized in this research were derived from 208,659 single-cell transcriptomes obtained from 60 individuals. Thirty-two clusters and 13 cell types were identified and visualized using the UMAP algorithm (Fig. 1A, B). Among them, B cells numbered 14,773, ranking within the top 5 (Fig. 1C). Ultimately, 279 B-cell marker genes of ESCA according to |log2FC| > 1 and adjusted *p*-value < 0.05. Among them, 274 genes can be matched in the TCGA-ESCA data, with 160 genes showing differential expression between cancer and normal samples in the TCGA-ESCA cohort (Table S1).

### Prognostic Model Development and Validation

**Fig. 2:**
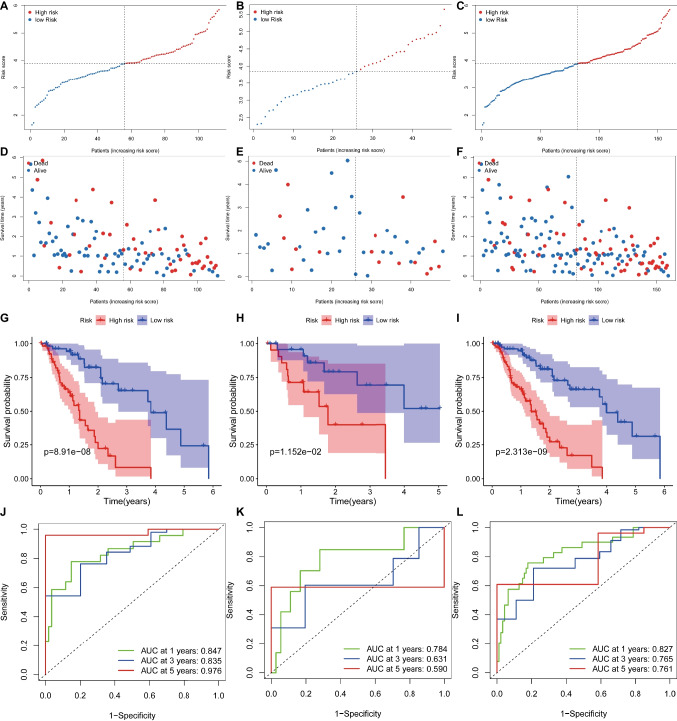
Construction and validation of prognostic models. **A**–**C** Distribution of BRGrisk score in training cohort, testing cohort, and whole TCGA-ESCA cohort, respectively. **D**–**F** Scatter plot of the OS of each patient in the training cohort, testing cohort, and whole TCGA-ESCA cohort, respectively. **G**–**I** The Kaplan-Meier curves in the training cohort, testing cohort, and whole TCGA-ESCA cohort, respectively. **J**–**L** The AUC at 1, 3, and 5 years of prognostic models in the training cohort, testing cohort, and whole TCGA-ESCA cohort, respectively

Using univariate Cox regression analysis, we identified 18 B-cell marker genes significantly associated with prognosis within the training cohort (*p* < 0.05) (Table S2). Through LASSO analysis, we further refined the selection to ten genes based on optimal lambda values and corresponding coefficients. Utilizing these genes, we formulated the BRGrisk model as follows: BRGrisk = −0.235 × CD38 + −0.129 × AHNAK + 0.532 × DSTN + 0.289 × DNAJB1 + −0.207 × ANXA5 + 0.122 × CD3D + 0.117 × CXCL8 + −0.055 × MT1E + 0.183 × CD7 + 0.491 × CCL3. The patients were then classified into high-risk and low-risk groups based on the median BRGrisk value. Visualizing BRGrisk scores on a scatter plot revealed a proportional correlation with decreasing OS and escalating mortality (Fig. 2A, D). Subsequently, we subjected the model to prognostic evaluation. The high-risk group exhibited significantly shorter survival than the low-risk group (*p* < 0.001) (Fig. 2G). The area under the curve (AUC) values for 1-, 3-, and 5-year survival in the training cohort were 0.847, 0.835, and 0.976, respectively (Fig. 2J). To ascertain the model’s robustness, we performed identical analyses on the test cohort (Fig. 2B, E) and all TCGA-ESCA samples (Fig. 2C, F). The test cohort results revealed superior OS for the low-risk group compared to the high-risk group (*p* = 0.012) (Fig. 2H), with AUCs of 0.784, 0.631, and 0.590 for 1-, 3-, and 5-year survival, respectively (Fig. 2K). Similarly, analysis of all TCGA-ESCA samples demonstrated better OS for the low-risk group (*p* < 0.001) (Fig. 2I), accompanied by AUCs of 0.827, 0.765, and 0.761 for 1-, 3-, and 5-year survival, respectively (Fig. 2L). The collective outcomes consistently indicated the model’s strong predictive capability.

### Construction and Assessment of the Nomogram Survival Model

**Fig. 3:**
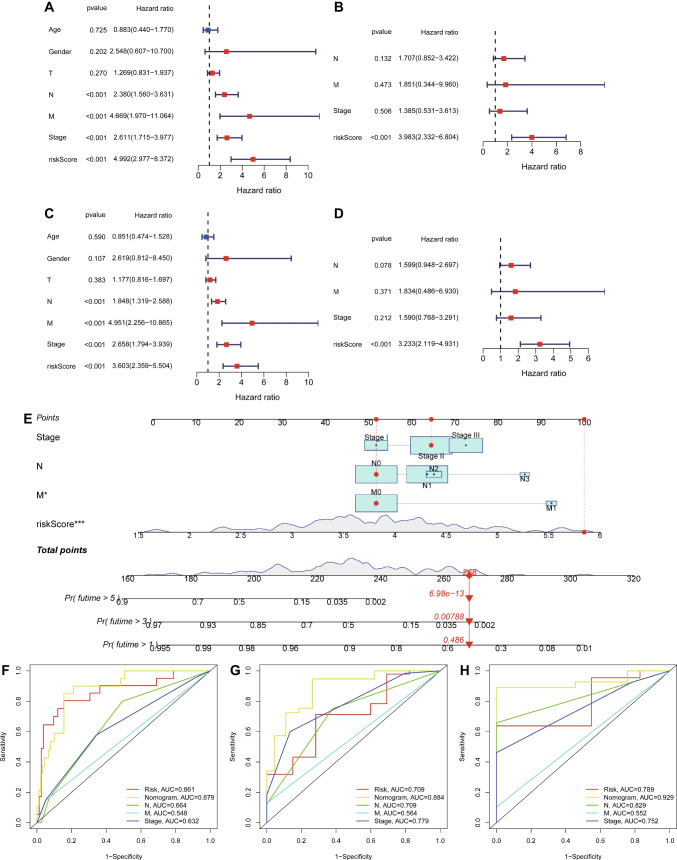
Predictive nomogram. **A** Univariate analysis of clinicopathologic features and BRG risk in the training cohort. **B** Multivariate analysis of clinicopathologic features and BRG risk in the training cohort. **C** Univariate analysis of clinicopathologic features and BRG risk in the entire TCGA-ESCA cohort. **D** Multivariate analysis of clinicopathologic features and BRG risk in the entire TCGA-ESCA cohort. **E** Nomogram for predicting the survival of ESCA patients. **F**–**H** ROC curves for clinicopathologic features and BRG risk, depicting 1-, 3-, and 5-year survival, respectively

To ascertain the potential independent prognostic role of BRGrisk, we performed both univariate and multivariate Cox regression analyses in the training cohort. Our findings from the univariate Cox regression analysis unveiled BRGrisk as a significant risk factor, with a hazard ratio (HR) of 4.992 and a 95% confidence interval (CI) spanning from 2.977 to 8.372 (*p* < 0.001, Fig. 3A). Additionally, in a multivariate analysis adjusted for other potential confounding factors, BRGrisk sustained its status as an independent prognostic factor for ESCA patients, yielding an HR of 3.983 and a 95% CI of 2.332 to 6.804 (*p* < 0.001, Fig. 3B). These were further confirmed in all TCGA-ESCA patients (*p* < 0.001, Fig. 3C, D). In order to provide a practical prognostic tool, we developed a nomogram model by integrating N stage, M stage, overall Stage, and BRGrisk. This nomogram aimed to estimate the probabilities of 1-, 3-, and 5-year OS for ESCA patients within the TCGA cohort (Fig. 3E). The model’s predictive accuracy was verified through the ROC curves; the AUC values demonstrated the nomogram’s efficacy in accurately predicting the 1-, 3-, and 5-year survival outcomes of ESCA patients (Fig. 3F–H).

### Gene Set Enrichment Analysis

**Fig. 4:**
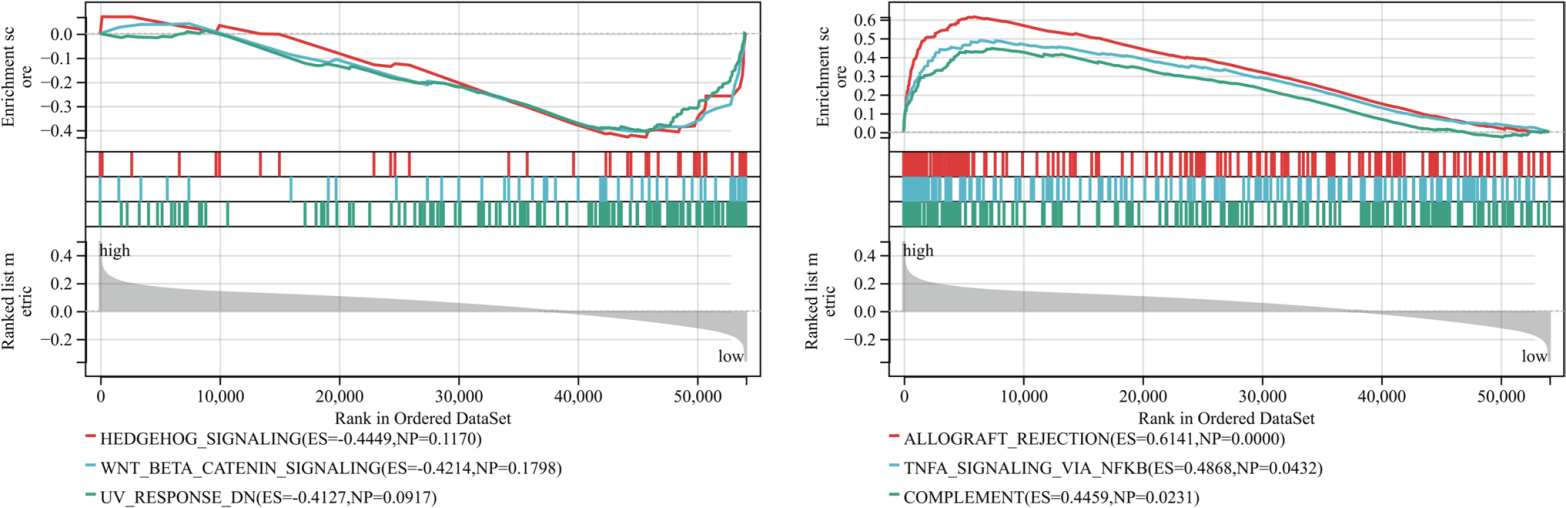
Biological characterization of of high- and low-BRGrisk groups. The GSEA pathway enrichment analysis in low- (left) and high- (right) ERS groups

The outcomes of the GSEA demonstrated a predominant enrichment of cancer-related pathways within the high-risk group of the training cohort. These pathways encompassed processes such as ALLOGRAFT_REJECTION, TNFA_SIGNALING_VIA_NFKB, and COMPLEMENT, among others (Fig. 4). The high-risk group primarily shows enrichment in HEDGEHOG_SIGNALING, WNT_BETA_CATENIN_SIGNALING, and UV_RESPONSE_DN pathways.

### Utilizing the BRGrisk Gene Signature for Assessing Tumor Immune Characteristics

**Fig. 5:**
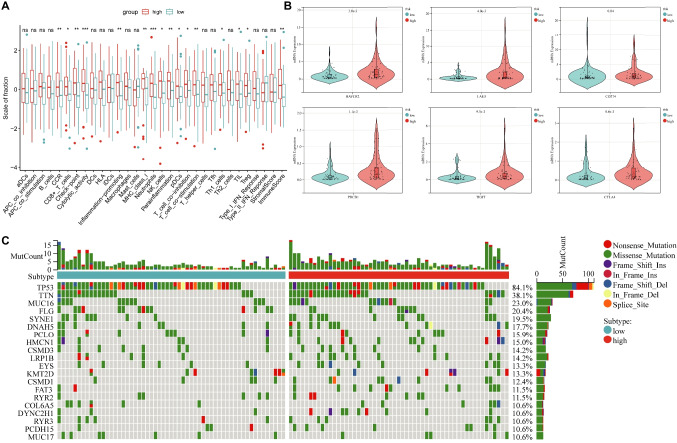
Immunological characterization of high- and low-BRGrisk groups. **A** The expression of immune function between different groups. **B** The expression levels of immune checkpoint genes between different groups. ns, not significant, *p < 0.05; **p < 0.01; ***p < 0.001

Employing the ssGSEA, we unveiled distinct levels of immune cell infiltration, immune functions, and immune scores that differentiated the high- and low-BRGrisk groups (Fig. 5A). Notably, the high-BRGrisk group demonstrated an enrichment of CCR, CD8+ T cells, checkpoint pathways, cytolytic activity, inflammation-promoting, and MHC class I (Fig. 5A). Additionally, immune checkpoint genes, including HAVCR2, LAG3, CD274, PDCD1, TIGIT, and CTLA4, exhibited significantly heightened expression in the high-BRGrisk group (Fig. 5B). Furthermore, the high-risk group exhibited a higher frequency of gene mutations (Fig. 5C).

### Drug Sensitivity Analysis

**Fig. 6:**
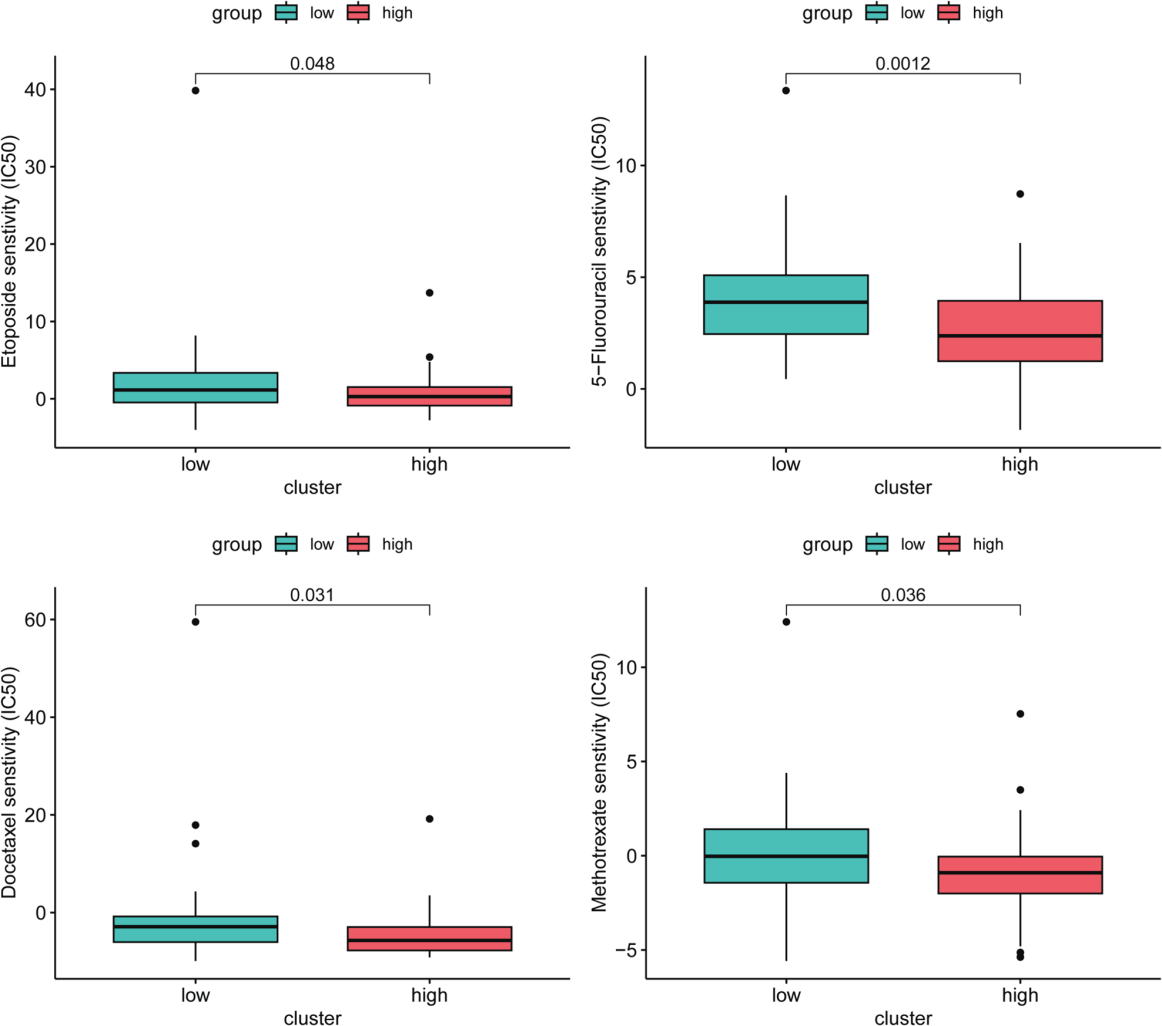
Drug sensitivity analysis of high- and low-BRGrisk groups. The boxplot of sensitivity of common chemotherapy drugs in different groups

In a more comprehensive assessment, we delved into the variation of IC50 levels for various chemotherapeutic drugs between the low-risk and high-risk groups (Fig. 6). Our findings unveiled that individuals classified within the low-risk group exhibited higher IC50 values for a range of anticancer agents, including etoposide, 5-fluorouracil, docetaxel, and methotrexate. These results strongly suggest that the potential of the BRGrisk model as a predictive tool for aiding in the choice of suitable anticancer therapies is significant, and individuals classified as low-risk might exhibit heightened responsiveness to anticancer agents.

## Discussion

The body of evidence suggests that ESCA is characterized by significant heterogeneity, manifesting complex interactions between tumor cells and immune cells [12, 27]. In the context of the burgeoning field of tumor immunotherapy, investigations into the tumor immune microenvironment have proliferated, revealing diverse immune cell subtypes and their pertinent functions. Despite the absence of direct tumor-killing capabilities, B cells are pivotal in their roles as antigen-presenting cells. Furthermore, B cells exert influence on tumor cells through the secretion of antibodies and cytokines [28, 29]. In the present investigation, we conducted scRNA-seq analysis to investigate the B-cell marker genes within the context of ESCA. Subsequently, utilizing the training cohort, we established a prognostic signature. The efficacy of this signature was subsequently assessed using both the test cohort and the entirety of TCGA-ESCA samples, further validating its predictive potential. Furthermore, our analysis revealed elevated levels of immune scores, immune cell infiltration, immune checkpoint expression, and somatic mutations within the high-risk group.

In this study, the prognostic signature consisted of ten B-cell marker genes, namely CD38, AHNAK, DSTN, DNAJB1, ANXA5, CD3D, CXCL8, MT1E, CD7, and CCL3. There are reports indicating that most of these genes were correlated with the prognosis of cancer [30–36]. Additionally, some of these genes have been suggested as targeting agents for cancer treatment [37–43]. The effectiveness of the prognostic signature, which relies on the ten identified B-cell marker genes, was subsequently confirmed through validation in both the testing cohort and across all TCGA-ESCA samples. Our findings consistently aligned across both cohorts, underscoring the robustness and reproducibility of the signature. In addition, we developed a nomogram that graphically depicts and predicts patients’ probabilities of 1-, 3-, and 5-year survival. The ROC curves further substantiate the enhanced predictive accuracy of the nomogram. Thus, this nomogram holds the potential to guide the formulation of personalized assessment protocols for individuals with ESCA, facilitating optimal utilization of medical resources.

Given the pivotal role of the TME in influencing anti-tumor responses and ultimately impacting prognosis [44], our investigation delved into the correlation between BRGrisk and the TME. Initially, we noted a notable elevation in stromal scores within the high-risk group when contrasted with the low-risk group. Subsequently, an exploration of 29 immune cell infiltration levels unveiled a heightened presence of CD8+ T cells, NK cells, dendritic cells, and neutrophils in the high-risk group. This observation suggests that individuals in this group might be experiencing a more activated state of anti-tumor immune response. Moreover, immune checkpoint inhibitors (ICIs) represent a potential therapeutic avenue for ESCA [45]. Several randomized clinical trials have shown a substantial improvement in OS when using immunochemotherapy as the first-line treatment for metastatic ESCC [46, 47], as compared to doublet chemotherapy. Our findings further indicated elevated expressions of common immune checkpoint-related genes (HAVCR2, LAG3, CD274, PDCD1, TIGIT, and CTLA4) within the high-risk group, suggesting that immunotherapy might be particularly advantageous for this subgroup. In conclusion, individuals within the high-risk group exhibited enhanced immune cell infiltration and immune responsiveness, potentially rendering them more amenable to benefiting from immunotherapy interventions.

In order to offer more precise treatment guidance for ESCA, we conducted a drug sensitivity analysis across different risk groups. Our findings demonstrated that the low-risk group exhibited sensitivity to these anticancer drugs (etoposide, 5-fluorouracil, docetaxel, and methotrexate). These results offer valuable insights for the clinical selection of chemotherapy medications. In future studies, we aim to delve deeper into the clinical implications of these drug sensitivities among ESCA patients.

While this study has contributed fresh perspectives to advance the progression of novel therapies for ESCA, certain limitations warrant consideration. Primarily, the entirety of the cohort studies employed herein were retrospective, necessitating subsequent validation through prospective cohort investigations. Additionally, the confirmation of drug sensitivity necessitates further substantiation via cellular experimentation. Furthermore, the limited number of scRNA-seq samples and the volume of data accessible in public databases have constrained the comprehensiveness of the analyzed clinical and pathological parameters, potentially introducing biases. As such, the execution of multi-center, extensive-sample, prospective double-blind trials becomes imperative for substantiating these findings in the future.

## Conclusions

In summary, we have successfully formulated an innovative prognostic signature comprising ten B-cell marker genes through the integration of scRNA-seq and bulk RNA-seq data. Additionally, the BRGrisk exhibited significant correlations with both the TIME and drug sensitivity. Our study furnishes novel theoretical insights into the impact of B-cell marker genes on the prognosis and targeted therapy potential for patients with esophageal carcinoma.

### Supplementary Information

Below is the link to the electronic supplementary material.

Supplementary file1 (XLS 45 KB)

## Data Availability

No datasets were generated or analyzed during the current study.

## Abbreviations

TME: Tumor microenvironment
ESCA: Esophageal carcinoma
BRGrisk: A B-cell-related gene signature
scRNA-seq: Single-cell RNA sequencing
NK: Natural killer
TIME: Tumor immune microenvironment
TPM: Transcript per million
TCGA: The Cancer Genome Atlas
TISCH2: The Tumor Immune Single Cell Hub 2
BRGs: B-cell-related genes
OS: Overall survival
LASSO: The least absolute shrinkage and selection operator
K-M: Kaplan-Meier
ROC: Time-dependent receiver operating characteristic
ssGSEA: A single-sample gene set enrichment analysis
AUC: Area under the curve
HR: Hazard ratio
CI: Confidence interval
ICIs: Immune checkpoint inhibitors
